# Supplementary material for: The RNF214-TEAD-YAP signaling axis promotes hepatocellular carcinoma progression via TEAD ubiquitylation
Source: Nat Commun. 2024 Jun 11;15:4995. doi: 10.1038/s41467-024-49045-y (PMC11167002; doi:10.1038/s41467-024-49045-y)
Supplement: Supplementary file 1 — Supplementary Information [file 41467_2024_49045_MOESM1_ESM.pdf]

The RNF214-TEAD-YAP signaling axis promotes hepatocellular carcinoma progression via TEAD ubiquitylation

Mengjia Lin[1,2], Xiaoyun Zheng[2,3], Jianing Yan[4], Fei Huang[2], Yilin Chen[2,3], Ran Ding[2,3], Jinkai Wan[5,6], Lei Zhang[5], Chenliang Wang[2], Jinchang Pan[2], Xiaolei Cao[2,3], Kaiyi Fu[2], Yan Lou[7], Xin-Hua Feng[2,3,8], Junfang Ji[2,3,8], Bin Zhao[2,3,8], Fei Lan[5,6], Li Shen[2,9], Xianglei He[10], Yunqing Qiu[1,7,*] & Jianping Jin[2,3,7,8,*]

Supplementary information

Supplementary Figures and Figure legends

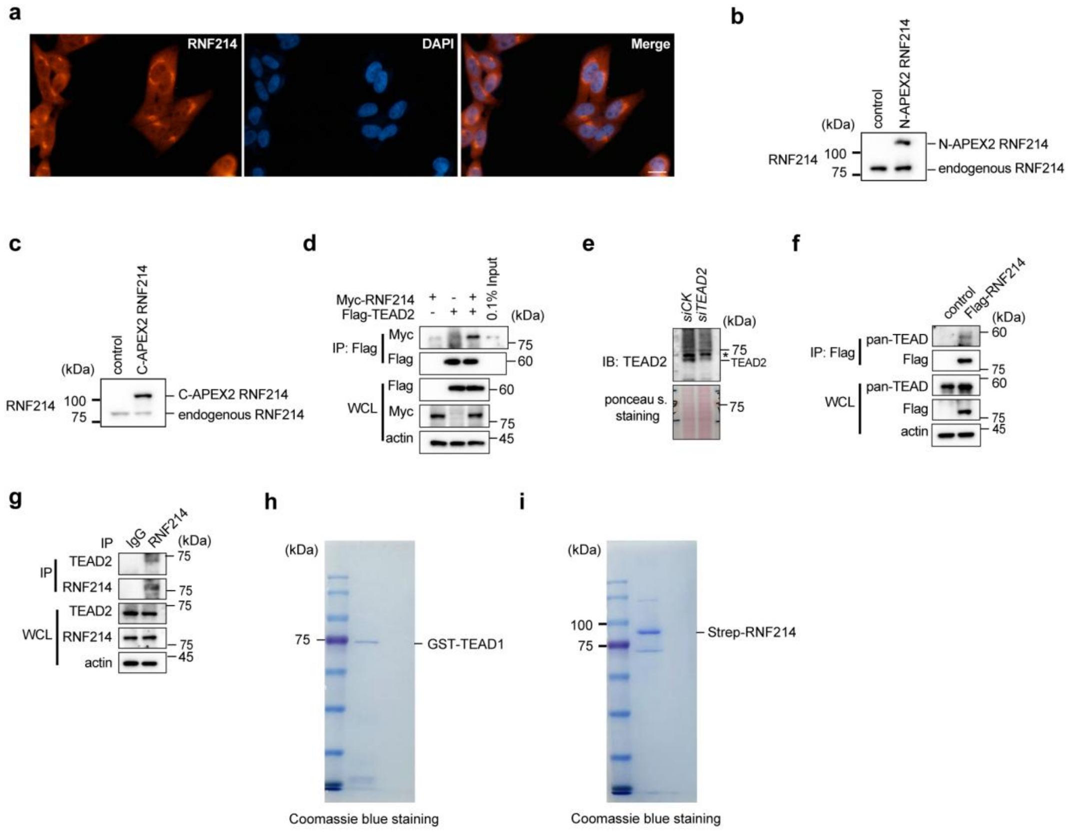

Supplementary Fig.1 RNF214 interacts with TEADs

a, RNF214 localizes in both the cytoplasm and the nucleus. Subcellular localization of RNF214 was detected using an immunofluorescence (IF) staining approach for endogenous RNF214 (red) along with DAPI for DNA staining (blue) in Hep3b cells. Scale bar, 20 μm. **b-c,** The expression levels of N-APEX2 RNF214 and C-APEX2 RNF214 fusion proteins in HLF cells. N-APEX2 or C-APEX2 fused RNF214 proteins

were stably expressed in HLF cells using a lentivirus infection method. Blank HLF cells were indicated as control. **d,** Association of RNF214 with TEAD2 in HEK293T cells. 0.1% input meant 0.1% of whole cell lysates which were used for IP. **e**, Knockdown of *TEAD2* in HEK293A cells. HEK293A cells were transfected with *siTEAD2* oligos and expression of TEAD2 was detected using Western blotting. '*' indicates non-specific band and ponceau s. staining was employed for loading control. **f,** Flag-RNF214 interacts with endogenous pan-TEAD in Hep3b cells. Hep3b cells were treated using 1 μM nocodazole for 15 mins and Flag-RNF214 was enriched using Flag-conjugated agarose. **g**, Endogenous TEAD2 interacts with RNF214 in HLF cells. RNF214 was purified by home-made RNF214 antibody (J044) from HLF cells, which were treated with 1 μM nocodazole for 15 mins. IgG antibody was used as the negative control. **h,** GST-tagged TEAD1 was purified using the bacteria system as shown using Coomassie blue staining. **i,** Strep-tagged RNF214 was purified using baculovirus-insect cell expression system and eluted by D-desthiobiotin as shown using Coomassie blue staining. All experiments in the figures were repeated at least twice. Source data are provided as a Source Data file.

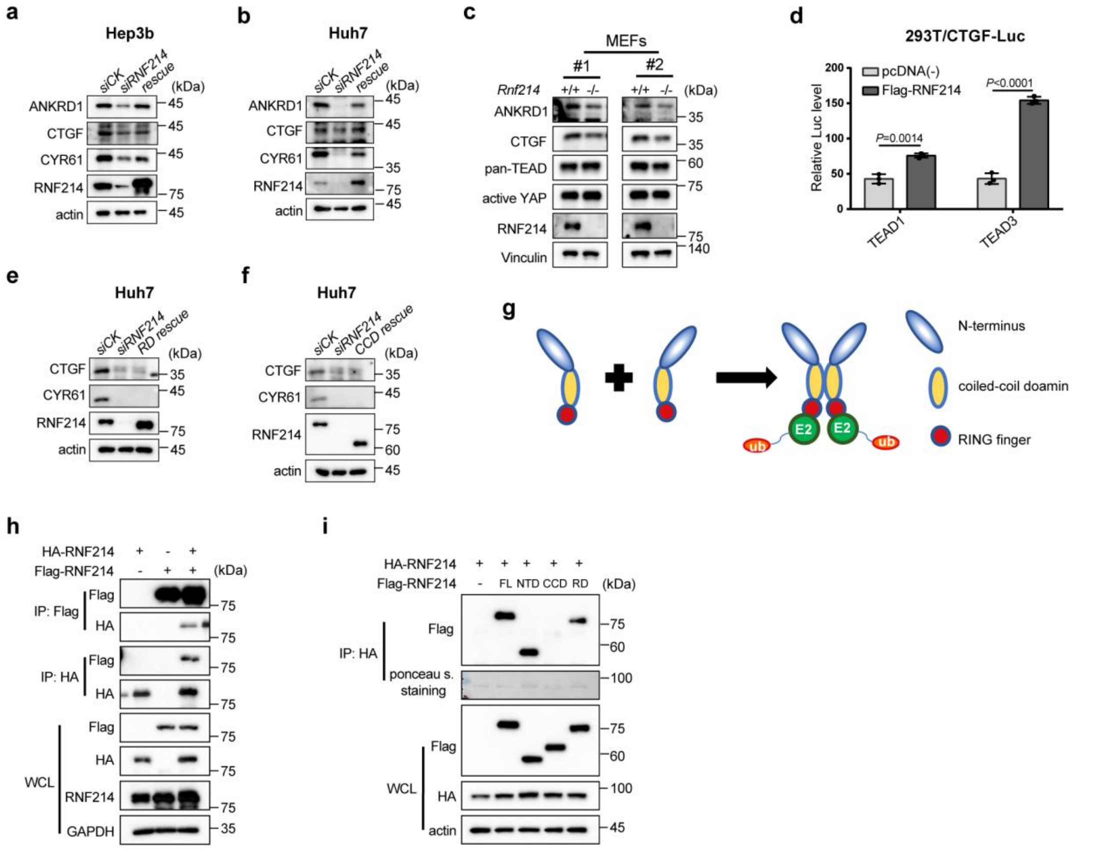

Supplementary Fig.2 RNF214 promotes Hippo-regulated transcription

a, RNF214 promotes the expression of TEADs target genes in Hep3b cells. *RNF214* was knocked down using siRNA and siRNA-resistant cDNA of *RNF214* was put back to *RNF214*-knockdown cells using lentivirus infection method to rescue reduced expression of TEADs target genes. Expressions of TEADs target proteins were detected using Western blotting. **b,** RNF214 promotes the expression of TEADs target genes in Huh7 cells. **c,** Western blotting of TEADs target proteins in MEF cells. Two independent pairs of MEF cells were isolated from 13.5 days' mouse embryos of two separated litters. **d,** RNF214 promotes TEAD1 and TEAD3 transcriptional activities. Myc-TEAD1 or HA-TEAD3 was co-transfected into HEK293T cells with CTGF-luc reporter, YAP and RNF214. Data are presented as mean ± SD. *P* values were calculated using two-sided unpaired Student's t-test; n=3 biologically independent samples. **e-f,** Western blotting of TEADs target genes in Huh7 cells. **g,** Schema showing oligomerization of RNF214. N-terminus sequence of RNF214, blue; coiled-coil domain of RNF214, yellow; RING finger of RNF214, red; a ubiquitin conjugating enzyme, E2 (green); ubiquitin, Ub (orange). **h,** RNF214 forms oligomer. HEK293T cells were transfected with Flag-RNF214 and HA-RNF214 plasmids. co-IP and immunoblotting were performed using anti-Flag or anti-HA antibodies as indicated. **i,** RNF214 self-associates through the coiled-coil domain. HEK293T cells were transfected with HA-RNF214 and full-length Flag-RNF214 or its mutants as indicated. FL: full length; NTD: N-terminal deletion; CCD: deletion of the coiled-coil domain; RD: RING finger deletion. Experiments in figures (**a, b, c, e, f, h, i)** were repeated twice. Source data are provided as a Source Data file.

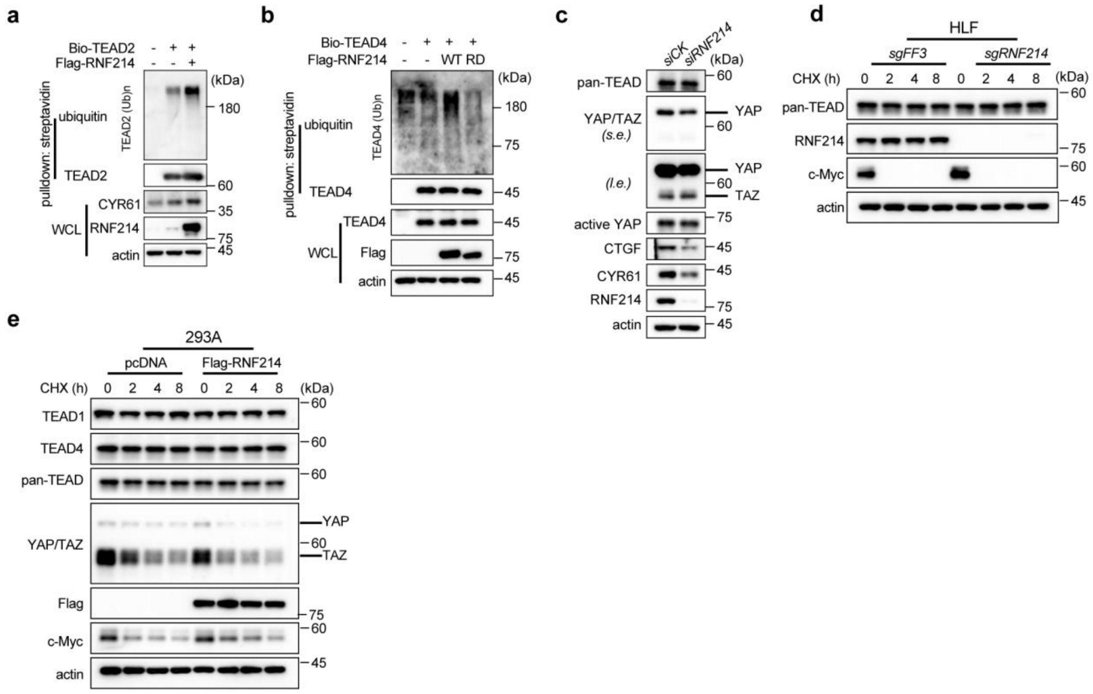

Supplementary Fig.3 RNF214 promotes nonproteolytic polyubiquitylation of TEADs

a, RNF214 promotes TEAD2 ubiquitylation. A biotinylated Avi-tagged TEAD2 (Bio-TEAD2) was expressed in Huh1 cells. Flag-RNF214 was transfected into the same Huh1 cells and biotin (2 μg/mL) was added to the culture medium overnight before cell harvesting. Biotinylated-TEAD2 proteins were pulled down using Streptavidin beads under a denaturing buffer condition. Ubiquitylated TEAD2 proteins were detected using an anti-ubiquitin antibody. **b,** The RD mutation of RNF214 reduces its ubiquitylation activity towards TEAD4. Flag-RNF214 WT or the RD mutant was transfected into HLF cells expressing a biotinylated Avi-TEAD4 (Bio-TEAD4). **c,** Depletion of *RNF214* has no effect on protein levels of TEADs and YAP/TAZ. Hep3b cells were transfected with *siRNF214* and protein samples were harvested for Western blotting using indicated antibodies. **d,** *RNF214* knockout does not affect the protein stability of TEADs in HLF cells. *RNF214* knockout or control HLF cells were treated using cycloheximide (CHX 20 μg/mL) for 0, 2, 4 or 8 hours before harvesting. c-Myc was employed as a positive control for CHX chase experiments. **e,** TEADs are stable proteins in RNF214-overexpressed HEK293A cells. HEK293A cells were transfected with pcDNA or Flag-RNF214 and then were treated using cycloheximide (CHX 20 μg/mL) for 0, 2, 4 or 8 hours before harvesting. Experiments in these figures were repeated twice. Source data are provided as a Source Data file.

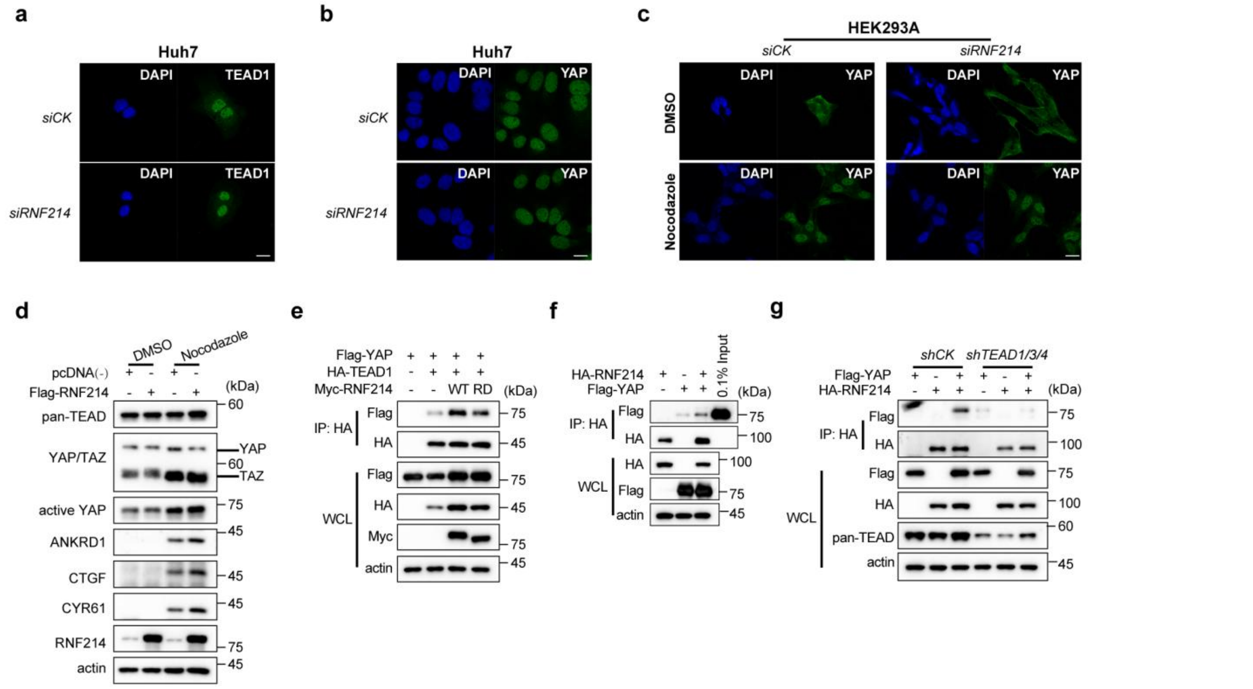

Supplementary Fig.4 RNF214 enhances the interaction between TEADs and YAP

a-b, RNF214 has no effect on nuclear localizations of TEAD1 and YAP. Subcellular localizations of TEAD1 and YAP were detected through IF approach for endogenous TEAD1 (green) or YAP (green) along with DAPI for DNA staining (blue) in Huh7 cells. Cells were fixed 96 hours after siRNF214 transfection. Scale bar, 20 μm. **c,** RNF214 does not affect cytoplasmic-nuclear shuttling of endogenous YAP. HEK293A cells were treated with 1 μM nocodazole for 1 hour after endogenous *RNF214* was depleted using *siRNF214* oligos. The cytoplasmic-nuclear shuttling of endogenous YAP was observed using an IF method. Scale bar, 20 μm. **d,** RNF214 Overexpression has little effect on protein levels of YAP/TAZ and YAP activity in HEK293A cells. HEK293A cells were transfected with Flag-RNF214 for 24 hours and then treated with 1 μM nocodazole for 2 hours. Protein samples were harvested for Western blotting using indicated antibodies. **e,** RNF214 promotes the interactions between YAP and TEAD1. HEK293T cells were transfected with Flag-YAP, HA-TEAD1 and Myc-RNF214 WT or RD mutant plasmids. **f-g,** RNF214 interacts with YAP weakly. HEK293T cells were transfected with HA-RNF214 and Flag-YAP, and co-IP and Western blotting were employed using antibodies as indicated. Experiments in these figures were repeated twice. Source data are provided as a Source Data file.

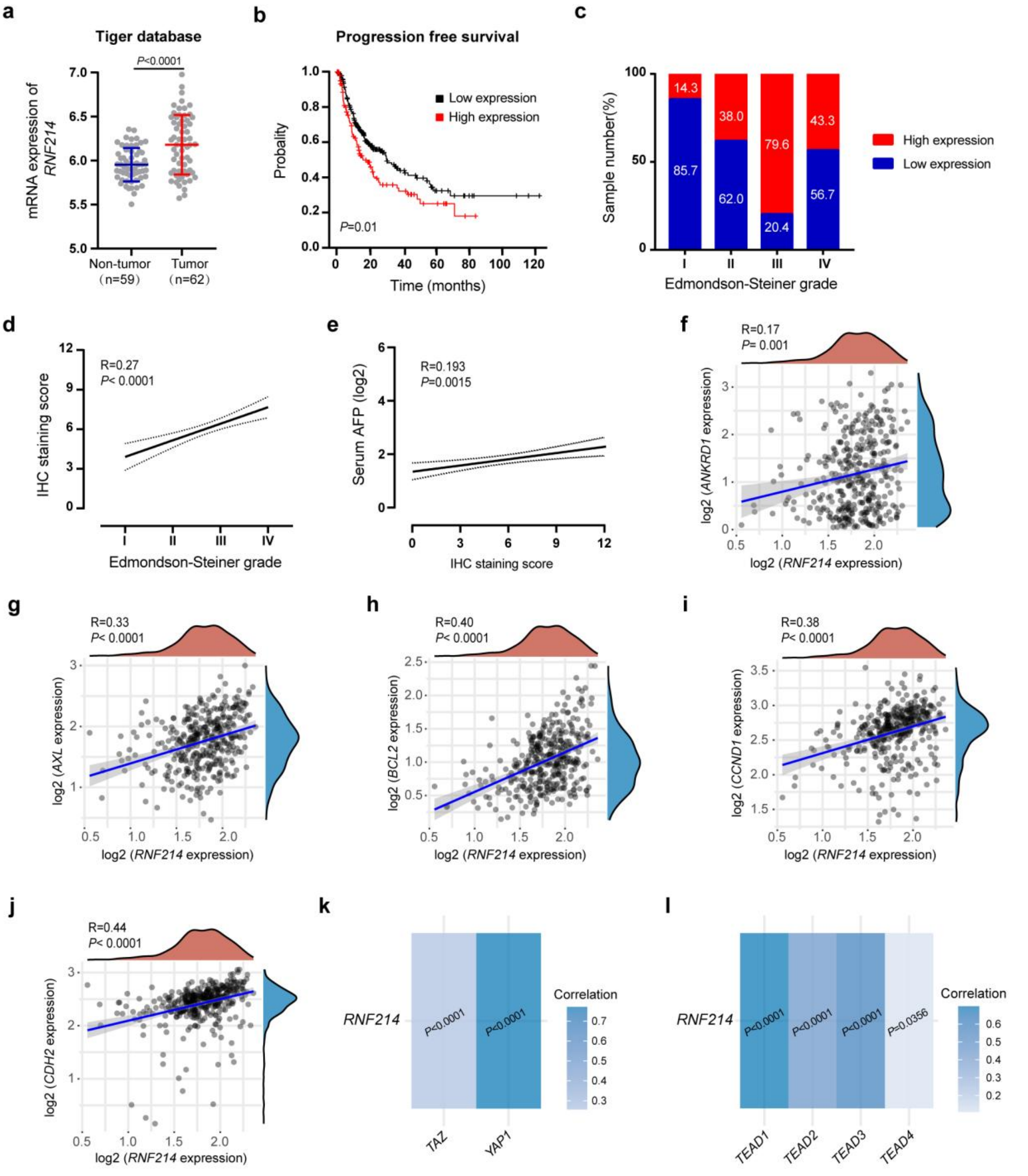

Supplementary Fig.5 Overexpression of RNF214 correlates with poor prognosis in HCC

a, Bioinformatic analysis of *RNF214* mRNA levels in HCC. mRNA expression levels from Tiger database were analyzed using two-sided unpaired Student's t-test. n=59 in the non-tumor group (blue); n=62 in the tumor group (red). Data are presented as mean ± SD. **b,** Kaplan-Meier survival curves of progression free survival based on *RNF214* mRNA expression levels from the TCGA database. The figure was produced from Kaplan-Meier Plotter. n=249 in the low expression group (black); n=121 in the high expression group (red). **c-d,** Spearman's correlation analysis between RNF214 and Edmondson-Steiner grade in tumor tissues from 275 patients with HCC. RNF214 (high), IHC score ≥ 6 (red)； RNF214 (low), IHC score < 6 (blue). Edmondson-Steiner Grade Ⅰ (RNF214 high, n=2; RNF214 low, n=12); Grade Ⅱ (RNF214 high, n=30; RNF214 low, n=49); Grade Ⅲ (RNF214 high, n=121; RNF214 low, n=31);Grade Ⅳ

(RNF214 high, n=13; RNF214 low, n=17). **e,** Spearman's correlation analysis between RNF214 and serum AFP levels (n=275). **f-j,** Expression level of *RNF214* is positively correlated with YAP/TAZ-TEAD target genes (e.g., *ANKRD1, AXL, BCL2, CCND1,* and *CDH2*) in liver cancer patients, as analyzed through Spearman's correlation analysis from TCGA database (n=371). **k-l,** Expression level of *RNF214* is positively correlated with *YAP*, *TAZ*, and *TEAD1-4*, as analyzed using TCGA database (n=371) through Spearman's correlation analysis. Source data are provided as a Source Data file.

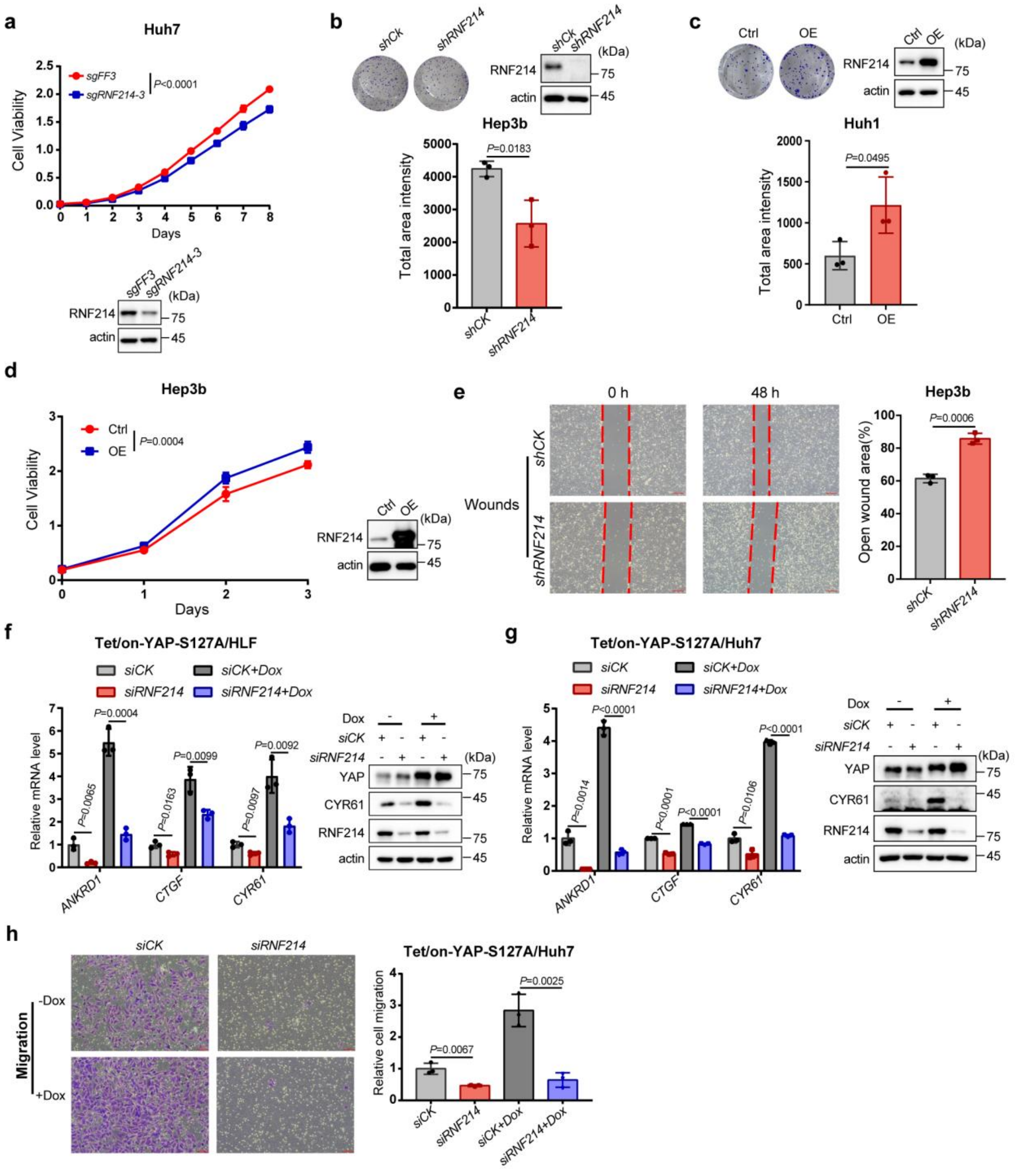

Supplementary Fig.6 RNF214 is critical for HCC tumorigenesis

a, Proliferation in *RNF214* knockout Huh7 cells. The cell viability rates of *RNF214* knockout HLF cells were quantified using CCK8 assay. n=5 biologically independent samples. **b,** Colony formation assays in *RNF214*-silenced Hep3b cells. The cells were incubated for 7 days. The total area intensity of colonies was measured and shown in the bar graph. n=3 biologically independent samples. **c,** Colony formation assay in Huh1 cells. Huh1 cells, with relatively low expression of RNF214, were used to overexpress exogenous RNF214 stably (OE) and subjected to colony formation assay. Huh1 cell lines were incubated for 12 days. The total area intensity of colonies was measured and shown in the bar graph. n=3 biologically independent samples. **d,** Cell viability analysis in RNF214-overexpressed Hep3b cells. Exogenous RNF214 was stably overexpressed in Hep3b cells using a lentivirus infection approach. n=5

biologically independent samples. **e,** Wound-healing assays in Hep3b cells. Microscopic images were shown at 0 and 48 hours (4x magnification). The open wound area (%) was measured and shown in the bar graph. Scale bar, 250 μm. n=3 biologically independent samples. **f-g,** Silencing *RNF214* dampened the S127A YAP-induced gene expression. HLF or Huh7 cells were stably introduced with the YAP-S127A mutant through the tetracycline-inducible gene expression system (Tet/on-YAP-S127A). The cell lines were then transfected with *siRNF214* oligos and 25 ng/ml Dox was added to the culture medium after 12 hours to induce the expression of YAP-S127A. n=3 biologically independent samples. **h,** Migration assay in the Tet/on-YAP-S127A Huh7 cell line. The Tet/on-YAP-S127A Huh7 cells were transfected with *siRNF214* and 25 ng/ml Dox was added to the culture medium after 12 hours. 48 hours post transfection, cells were plated in transwell chambers (with indicated Dox) for another 48 hours before analysis. Scale bar, 100 μm. n=3 biologically independent samples. Data are presented as mean ± SD. *P* values were calculated using two-sided unpaired Student's t-test from independent samples. Source data are provided as a Source Data file.

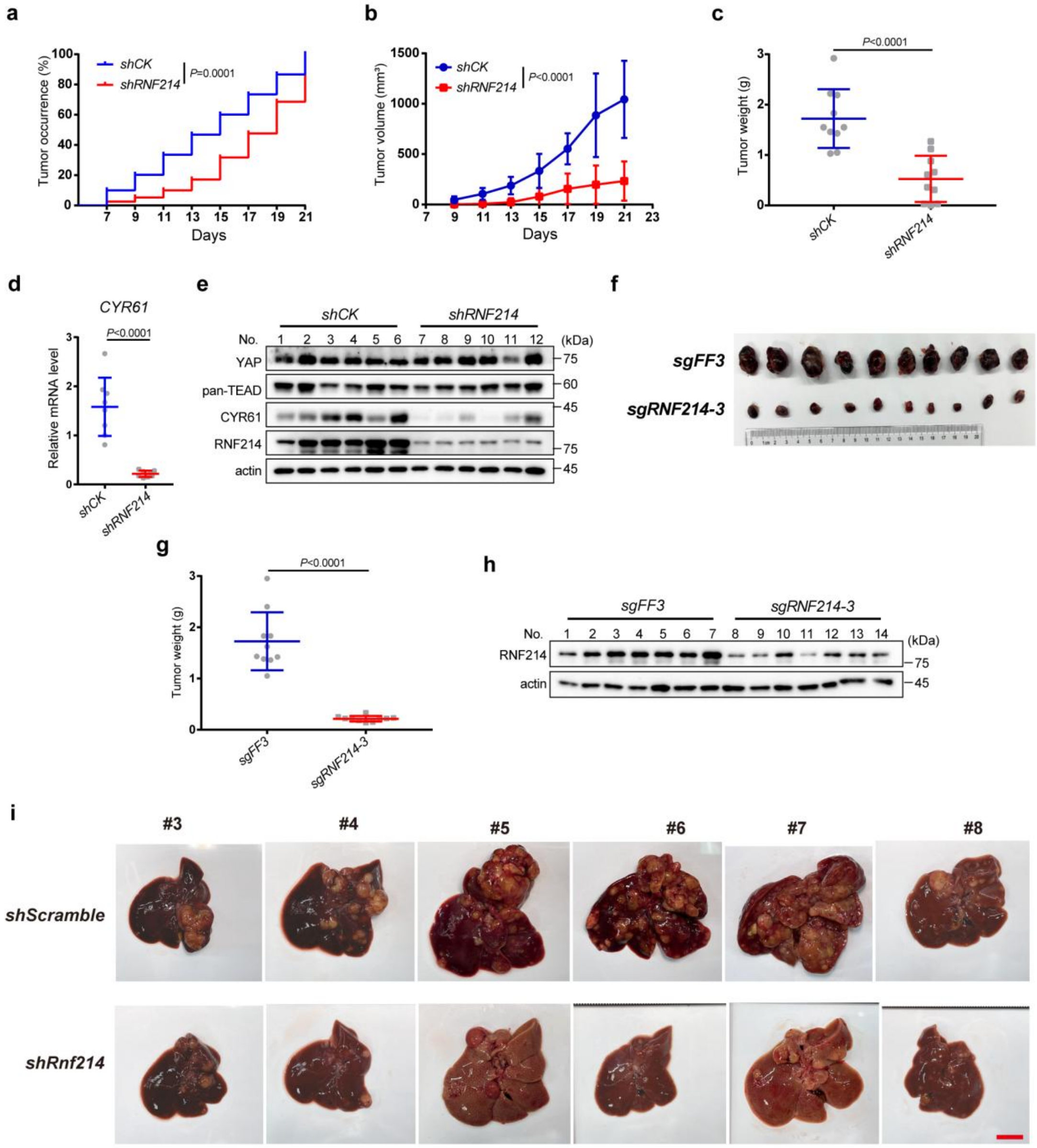

Supplementary Fig.7 RNF214 contributes to tumorigenesis in HCC

a-c, *RNF214* knockdown inhibited HCC tumor growth in subcutaneous xenograft model. The log-rank test and Cox regression analysis were used for tumor occurrence (**a**). Data are presented as mean ± SD; *P* values were calculated using two-sided unpaired Student's t-test (**b,c**). n=10 mice per group. **d-e,** mRNA and protein levels of CYR61 in *RNF214*-silenced tumors. The dissected tumors were subjected to qRT-PCR analysis (n=8) and Western blotting (n=6). Data are presented as mean ± SD. *P* values were calculated using two-sided unpaired Student's t-test. **f-h,** *RNF214* knockout inhibited HCC tumor growth in subcutaneous xenograft model. Control or *RNF214* knockout Huh7 cells with Matrigel were injected subcutaneously into 5-week-old male BALB/c nude mice. 21 days after cell implantation, tumors were dissected and photographed (**f**).Tumor weight (**g**) was recorded and the dissected tumors were subjected to Western blotting (n=7) (**h**). n=10 tumors for each group.

Data are presented as mean ± SD; *P* values were calculated using two-sided unpaired Student's t-test (**g**). **i,** Depletion of *Rnf214* suppressed tumor formation. Mice were sacrificed 120 days after injection and livers were pictured. Six representative livers in each cohort were shown. Scale bar, 1 cm. Source data are provided as a Source Data file.

Supplementary Table 1. Targeting sequences of siRNAs, shRNAs or gRNAs.

Name	Targeted Sequences
sgRNF214-1 (Homo sapiens)	GTCTGAGGTTGCTGGTGTTG
sgRNF214-2 (Homo sapiens)	GAATAACAGAAATGTCCATT
sgRNF214-3 (Homo sapiens)	GAGCACTCAGAGCAGAATCC
sgRNF214-4 (Homo sapiens)	GCCTTAAGAGACCGAGTGAC
shRNF214 (Homo sapiens)	CCATCCTAAAGGAAGGTAACA
shRnf214-1 (Mus musculus)	CGGGACCAGTTTAATAGTCAT
shRnf214-2 (Mus musculus)	GCGAGAAGAAACCAAGAAGAA
siCK (control siRNA)	GAUCCGCAGCGACAUCAACCU
siRNF214 (Homo sapiens)	CAAAUCCCUACUCCCACUUUA
siTEAD2 (Homo sapiens)	GUGGUGAAUUUCUUGCACA

Supplementary Table 2. Primers used for qRT-PCR analysis.

Name	Primer Sequences (5'-3')
ANKRD1 (Homo sapiens)	CACTTCTAGCCCACCCTGTGA<br>CCACAGGTTCCGTAATGATTT
CTGF (Homo sapiens)	CCAATGACAACGCCTCCTG<br>TGGTGCAGCCAGAAAGCTC
CYR61 (Homo sapiens)	GGGCTGGAATGCAACTTCG<br>GGCGCCATCAATACATGTGC
GAPDH (Homo sapiens)	AGGGCTGCTTTTAACTCTGGT<br>CCCCACTTGATTTTGGAGGGA

Supplementary Table 3. Reagents

Reagents	Source	Identifier
RNF214 Polyclonal, 1:1000 for WB	Sino Biological	202826-T38
YAP (63.7),1:1000 for WB and 1:100 for IF	Santa Cruz	sc-101199
YAP/TAZ (D24E4), 1:1000 for WB	Cell signaling technology	8418
pan-TEAD (D3F7L), 1:1000 for WB	Cell signaling technology	13295
TEAD1 (31/TEF-1), 1:500 for WB and 1:100 for IF	BD Biosciences	610922
TEAD2 Polyclonal, 1:500 for WB	Proteintech	21159-1-AP
TEAD3 Polyclonal, 1:500 for WB	Abcam	ab138246
TEAD4 (5H3) , 1:1000 for WB	Abcam	ab58310
ANKRD1Polyclonal, 1:1000 for WB	Proteintech	11427-1-AP
CTGF (E-5), 1:300 for WB	Santa Cruz	sc-365970
CYR61 (A-10), 1:500 for WB	Santa Cruz	sc-374129
CYR61 Polyclonal, 1:1000 for WB	Proteintech	26689-1-AP
Ubiquitin (P4D1), 1:1000 for WB	Cell signaling technology	3936
GAPDH (AC002), 1:3000 for WB	ABclonal	AC002
Actin (AC026), 1:200000 for WB	ABclonal	AC026
Flag (M2), 1:1000 for WB	Sigma	F3165
HA Polyclonal, 1:1000 for WB	Proteintech	51064-2-AP
Myc (19C2), 1:1000 for WB	Abmart	M20002
Strep Polyclonal, 1:1000 for WB	Genescript	A00626
Vinculin Polyclonal, 1:3000 for WB	Sigma	V4139
Peroxidase-conjugated AffiniPure Goat Anti-Mouse IgG (H+L), 1:10000 for WB	Jackson ImmunoResearch	158323
Peroxidase-conjugated AffiniPure Goat Anti-Rabbit IgG (H+L), 1:10000 for WB	Jackson ImmunoResearch	156592
Normal Rabbit IgG	Calbiochem	D00016762

The home-made Anti-RNF214 antibody (J044) was raised in rabbits using peptide corresponding to amino acids 259-273 of human RNF214.
