# Supplementary material for: The RNF214-TEAD-YAP signaling axis promotes hepatocellular carcinoma progression via TEAD ubiquitylation
Source: Nat Commun. 2024 Jun 11;15:4995. doi: 10.1038/s41467-024-49045-y (PMC11167002; doi:10.1038/s41467-024-49045-y)
Supplement: Supplementary file 2 — Reporting Summary [file 41467_2024_49045_MOESM2_ESM.pdf]

nature portfolio

Corresponding author(s): Yunqing Qiu & Jianping Jin

Last updated by author(s): 2024/3/26

Reporting Summary

Nature Portfolio wishes to improve the reproducibility of the work that we publish. This form provides structure for consistency and transparency in reporting. For further information on Nature Portfolio policies, see our Editorial Policies and the Editorial Policy Checklist.

Statistics

For all statistical analyses, confirm that the following items are present in the figure legend, table legend, main text, or Methods section.

n/a	Confirmed	
☐	☒	The exact sample size (*n*) for each experimental group/condition, given as a discrete number and unit of measurement
☐	☒	A statement on whether measurements were taken from distinct samples or whether the same sample was measured repeatedly
☐	☒	The statistical test(s) used AND whether they are one- or two-sided<br>*Only common tests should be described solely by name; describe more complex techniques in the Methods section.*
☒	☐	A description of all covariates tested
☒	☐	A description of any assumptions or corrections, such as tests of normality and adjustment for multiple comparisons
☐	☒	A full description of the statistical parameters including central tendency (e.g. means) or other basic estimates (e.g. regression coefficient) AND variation (e.g. standard deviation) or associated estimates of uncertainty (e.g. confidence intervals)
☐	☒	For null hypothesis testing, the test statistic (e.g. *F*, *t*, *r*) with confidence intervals, effect sizes, degrees of freedom and *P* value noted<br>*Give P values as exact values whenever suitable.*
☒	☐	For Bayesian analysis, information on the choice of priors and Markov chain Monte Carlo settings
☒	☐	For hierarchical and complex designs, identification of the appropriate level for tests and full reporting of outcomes
☐	☒	Estimates of effect sizes (e.g. Cohen's *d*, Pearson's *r*), indicating how they were calculated

Our web collection on statistics for biologists contains articles on many of the points above.

Software and code

Policy information about availability of computer code

Data collection	Immunofluorescence were taken by LSM 710 (Zeiss) confocal microscopy. HE or IHC stained sections were scanned with digital section scanner (KF-FL-020). qRT-PCR analysis was performed by the SYBR green method (YEASEN) on Bio-Rad CFX96 system. The dual luciferase assay was performed using Dual Luciferase Reporter Assay System (Vazyme) and detected by the Spark Multimode Microplate readers (TECAN).The Mass Spec were finished on timsTOF Pro (Bruker), and the data were analyzed by PEAKS online. The KEGG pathway enrichment analysis was performed using 'clusterProfiler' R package.
Data analysis	Image J (version 1.52r) for image analysis, GraphPad Prism (version7) for statistical analysis

For manuscripts utilizing custom algorithms or software that are central to the research but not yet described in published literature, software must be made available to editors and reviewers. We strongly encourage code deposition in a community repository (e.g. GitHub). See the Nature Portfolio guidelines for submitting code & software for further information.

Data

Policy information about availability of data

All manuscripts must include a data availability statement. This statement should provide the following information, where applicable:

- Accession codes, unique identifiers, or web links for publicly available datasets
- A description of any restrictions on data availability
- For clinical datasets or third party data, please ensure that the statement adheres to our policy

All data are available in the main text or supplementary informations in the Source Data. Human data derived from the TCGA, Fanjia, and Tiger datasets were

provided in the source data. The statistical analysis of the overall survival was through the Human Protein Atlas website (https://www.proteinatlas.org/). The statistical analysis of the progression free survival was through the Kaplan Meier Plotter website (http://kmplot.com/analysis/). The Spearman's rank correlation analysis of the TCGA cohort was through the bioinformatic website (https://www.aclbi.com/static/index.html#/). The mass spectrometry data generated in this study have been deposited in the ProteomeXchange Consortium with the dataset identifier PXD049453.

Research involving human participants, their data, or biological material

Policy information about studies with human participants or human data. See also policy information about sex, gender (identity/presentation), and sexual orientation and race, ethnicity and racism.

Reporting on sex and gender	No significant association with gender was observed in the study.
Reporting on race, ethnicity, or other socially relevant groupings	No significant association with race, ethnicity and other socially relevant groupings was observed in the study.
Population characteristics	No significant association with age and gender was observed in the study.
Recruitment	Human HCC tissues and adjacent non-tumor tissues microarray chips were created in Department of Pathology, Zhejiang Provincial People's Hospital (China).
Ethics oversight	The study was approved by the Medical Ethic Committee of Zhejiang Provincial People's Hospital.

Note that full information on the approval of the study protocol must also be provided in the manuscript.

Field-specific reporting

Please select the one below that is the best fit for your research. If you are not sure, read the appropriate sections before making your selection.

☒ Life sciences ☐ Behavioural & social sciences ☐ Ecological, evolutionary & environmental sciences

For a reference copy of the document with all sections, see nature.com/documents/nr-reporting-summary-flat.pdf

Life sciences study design

All studies must disclose on these points even when the disclosure is negative.

Sample size	The number of mice used in each experimental group was based on prior experience with liver cancer animal models (PMID:35710796; PMID: 34996827). For in vivo experiments, sample size is of n=8-10. For in vitro experiments, we used sample sizes as listed in the corresponding figure legends or figures. We aimed for a number of at least three independent experiments to allow for Student's t test (two sided).
Data exclusions	No data was excluded from the analyses.
Replication	The experiments including qRT-PCR, luciferase reporter assay, cell viability, colony formation, wound-healing assay, cell migration and cell invasion were performed at least 3 times to make sure similar results. And all datas were confirmed to be reproducible.
Randomization	Animals were randomly assigned according to the standard procedure. For in vitro experiments, cells were prepared, treated , and processed randomly.
Blinding	For animal experiments, investigators were blinded to the group. For the other experiments, investigators were not blinded to the experimental groups during data collection and analysis, because no data point was excluded or selected and the investigator had to know the groups to perform the experiments.

Reporting for specific materials, systems and methods

We require information from authors about some types of materials, experimental systems and methods used in many studies. Here, indicate whether each material, system or method listed is relevant to your study. If you are not sure if a list item applies to your research, read the appropriate section before selecting a response.

Materials & experimental systems

n/a	Involved in the study
☐	☒ Antibodies
☐	☒ Eukaryotic cell lines
☒	☐ Palaeontology and archaeology
☐	☒ Animals and other organisms
☒	☐ Clinical data
☒	☐ Dual use research of concern
☒	☐ Plants

Methods

n/a	Involved in the study
☒	☐ ChIP-seq
☒	☐ Flow cytometry
☒	☐ MRI-based neuroimaging

Antibodies

Antibodies used

Anti-human/mouse RNF214 (202826-T38, Sino Biological,1:1000), anti-human/mouse YAP (63.7) (sc-101199, Santa Cruz/SC,1:1000 for WB and 1:100 for IF), anti-human/mousse YAP/TAZ (D24E4) (8418, Cell signaling technology/CST, 1:1000), anti-human/mouse pan-TEAD (D3F7L) (13295, CST,1:1000), anti-human/mouse TEAD1 (31/TEF-1) (610922, BD Biosciences, 1:500 for WB and 1:100 for IF), anti-human/mouse TEAD2 (21159-1-AP, Proteintech, 1:500), anti-human TEAD3 (ab138246, Abcam, 1:500), anti-human TEAD4 (5H3) (ab58310, Abcam, 1:1000), anti-human/mouse ANKRD1 (11427-1-AP, Proteintech, 1:1000), anti-human/mouse CTGF (E-5) (sc-365970, SC,1:300), anti-human CYR61 (A-10) (sc-374129, SC,1:500), anti-mouse CYR61(26689-1-AP, Proteintech,1:1000), anti-human/mouse Ubiquitin (P4D1) (3936, CST, 1:1000), anti-human/mouse GAPDH (AC002) (AC002, ABclonal. 1:3000), anti-human/mouse actin (AC026) (AC026, ABclonal,1:200000), Flag (M2) (F3165, Sigma, 1:1000), HA (51064-2-AP, Proteintech,1:1000), Myc (19C2) (Abmart, M20002, 1:1000), Strep (A00626, Genescript, 1:1000), anti-human/mouse Vinculin (V4139, Sigma,1:3000).

Validation

RNF214 antibody was validated by KO cell lines in figure 1a and 6a both in mouse and human cells. YAP antibody was validated previously (PMID: 37595580). YAP/TAZ antibody was validated previously (PMID: 36509758). pan-TEAD antibody was validated previously (PMID: 37277530). TEAD1 antibody was validated previously (PMID: 22863277). TEAD2 antibody was validated by KD cell lines in supplementary figure S1e. TEAD4 antibody was validated previously (PMID: 35115527). ANKRD1 antibody was validated previously (PMID: 32404936). CTGF antibody was validated previously (PMID: 37029927). CYR61 (Santa Cruz) antibody was validated previously (PMID: 25601475). CYR61 (Proteintech) antibody was validated by the company (https://www.ptgcn.com/products/CYR61-CCN1-Antibody-26689-1-AP.htm#publications). Ubiquitin antibody was validated previously (PMID: 37798264). GAPDH antibody was validated by the company (https://abclonal.com.cn/catalog/AC002). Actin antibody was validated by the company (https://abclonal.com.cn/catalog/AC026). Flag antibody was validated by the company (https://www.sigmaaldrich.cn/CN/zh/product/sigma/f3165). HA antibody was validated by the company (https://www.ptgcn.com/products/HA-tag-Antibody-51064-2-AP.htm). Myc antibody was validated by the company (http://www.ab-mart.com.cn/page.aspx?node=%2059%20&id=%20962). Strep antibody was validated by the company (https://www.genscript.com.cn/antibody/A00626-NWSHPQFEK_Antibody_pAb_Rabbit.html). Vinculin antibody was validated by the company (https://www.sigmaaldrich.cn/CN/zh/product/sigma/V4139).

Eukaryotic cell lines

Policy information about cell lines and Sex and Gender in Research

Cell line source(s)

HEK293T (CRL-11268) cells were from ATCC. HEK293A (ATCC, CRL-1573), Snu449 (ATCC, CRL-2234), and HL7702 (Chinese Academy of Sciences Cell bank, GNHu6) cells were from Dr. Bin Zhao. HepG2 (ATCC, HB-8055), Hep3b (ATCC, HB-8064), Huh7 (JCRB cell bank, JCRB 0403), Huh1 (JCRB cell bank, JCRB 0199), HLF (JCRB cell bank, JCRB 0405), and HLE (JCRB cell bank, JCRB 0404) cells were from Dr. Junfang Ji. Primary MEF cells were isolated from 13.5 days' mouse embryos and the sex is not under consideration.

Authentication

Cell lines were not authenticated.

Mycoplasma contamination

All cell lines tested negative for mycoplasma contamination.

Commonly misidentified lines
(See ICLAC register)

No such cell lines were used.

Animals and other research organisms

Policy information about studies involving animals; ARRIVE guidelines recommended for reporting animal research, and Sex and Gender in Research

Laboratory animals

5-week-old male BALB/c nude mice and 4-week-old male ICR mice used were obtained from Shanghai SLAC Laboratory Animal Company. Standard laboratory chow diet for mice were purchased from XieTong Biology (Cat#1010082) and the SPF grade animal room maintained humidity (45%-60%) with a 12 hours (7:00 a.m. -7:00 p.m.) light/dark cycle.

Wild animals

No wild animals involved in this study.

Reporting on sex

We used male mice for in vivo experiments. It has been consistent in the field to use male mice only, since it is much harder and takes much longer to achieve consistent results if using female mice.

Field-collected samples	This study didn't involve samples collected from field.
Ethics oversight	All animal experiments were strictly accordance with the recommendations approved by the Animal Ethics Committee of Zhejiang University. We used a humane protocol in our xenograft tumor growth assay with the endpoints of tumor volume less than 1500 mm3.

Note that full information on the approval of the study protocol must also be provided in the manuscript.

Plants

Seed stocks	No plants in this study.
Novel plant genotypes	No plants in this study.
Authentication	No plants in this study.
